# Potential of 2-Chloro-*N*-(4-fluoro-3-nitrophenyl)acetamide Against *Klebsiella pneumoniae* and In Vitro Toxicity Analysis

**DOI:** 10.3390/molecules25173959

**Published:** 2020-08-31

**Authors:** Laísa Cordeiro, Hermes Diniz-Neto, Pedro Figueiredo, Helivaldo Souza, Aleson Sousa, Francisco Andrade-Júnior, Thamara Melo, Elba Ferreira, Rafael Oliveira, Petrônio Athayde-Filho, José Barbosa-Filho, Abrahão Oliveira-Filho, Edeltrudes Lima

**Affiliations:** 1 Department of Pharmaceutical Science, Health Sciences Center, Federal University of Paraíba, 58033-455 João Pessoa, Paraíba, Brazil; hermes.dn@hotmail.com (H.D.-N.); pedrotrfigueiredo@gmail.com (P.F.); aleson_155@hotmail.com (A.S.); juniorfarmacia.ufcg@outlook.com (F.A.-J.); th.rmelo@outlook.com (T.M.); elbaferreira99@gmail.com (E.F.); jbarbosa@ltf.ufpb.br (J.B.-F.); edelolima@yahoo.com.br (E.L.); 2 Chemistry Department, Exact and Natural Sciences Center, Federal University of Paraíba, 58033-455 João Pessoa, Brazil; helivaldog3@gmail.com (H.S.); rfarias.quimica@gmail.com (R.O.); athayde-filho@quimica.ufpb.br (P.A.-F.); 3 Rural Health and Technology Center, Federal University of Campina Grande, 58708-110 Patos, Brazil; abrahao.farm@gmail.com

**Keywords:** 2-chloro-*N*-(4-fluoro-3-nitrophenyl)acetamide, *Klebsiella pneumoniae*, antibacterial, toxicity, minimum inhibitory concentration

## Abstract

*Klebsiella pneumoniae* causes a wide range of community and nosocomial infections. The high capacity of this pathogen to acquire resistance drugs makes it necessary to develop therapeutic alternatives, discovering new antibacterial molecules. Acetamides are molecules that have several biological activities. However, there are no reports on the activity of 2-chloro-*N*-(4-fluoro-3-nitrophenyl)acetamide. Based on this, this study aimed to investigate the in vitro antibacterial activity of this molecule on *K. pneumoniae*, evaluating whether the presence of the chloro atom improves this effect. Then, analyzing its antibacterial action more thoroughly, as well as its cytotoxic and pharmacokinetic profile, in order to contribute to future studies for the viability of a new antibacterial drug. It was shown that the substance has good potential against *K. pneumoniae* and the chloro atom is responsible for improving this activity, stabilizing the molecule in the target enzyme at the site. The substance possibly acts on penicillin-binding protein, promoting cell lysis. The analysis of cytotoxicity and mutagenicity shows favorable results for future in vivo toxicological tests to be carried out, with the aim of investigating the potential of this molecule. In addition, the substance showed an excellent pharmacokinetic profile, indicating good parameters for oral use.

## 1. Introduction

The *Klebsiella pneumoniae* bacteria, belonging to the Enterobacteriaceae family, are Gram-negative, facultative, immobile, and encapsulated anaerobes. It is one of the opportunistic pathogens of greatest clinical relevance, known to be associated with nosocomial infections. It is estimated that this species, alone, is responsible for approximately one third of all infections caused by Gram-negative bacteria in general, causing, for example, pneumonia, urinary tract infections, bacteremia, endocarditis, and liver abscesses. This pathogen is also involved in serious community infections, such as necrotizing pneumonia, pyogenic liver abscesses, and endogenous endophthalmitis [1,2].

In addition to being a highly prevalent species, the appearance of hypervirulent strains of *K. pneumoniae* has further expanded the number of people susceptible to infections by this species, increasing, for example, the number of cases in immunocompetent individuals. The species is also an important source of resistance to antibiotics, presenting a great capacity to acquire plasmids with genes that give them resistance to multiple antibacterials [2]. As explained by Paczosa and Mecsas [1], this pathogen is gaining more notoriety, since the increase in the number of serious infections and the growing scarcity of effective medications bring a great challenge in the treatment of these diseases.

In 2017, the World Health Organization (WHO) [3] released for the first time a list of resistant microorganisms that pose a threat to human health and for which there is a priority need for the development of new antibiotics. The experts used as a basis for the construction of this document criteria such as mortality, prevalence of resistance, and transmissibility. The list was divided into three levels of need for antibiotic development: critical, high, and medium. Compounding the critical group are Gram-negative bacteria, more specifically *Acinetobacter baumannii*, *Pseudomonas aeruginosa* and Enterobacteriaceae—which include *K. pneumoniae*—resistant to third-generation carbapenemas and cephalosporins [3].

One of the objectives of the list released by the WHO is to guide research strategies for new antibacterials. In recent years, experts have already warned of the difficulty in combating Gram-negatives, especially [4]. Quinolones, discovered in 1962, were the last new class of antibiotic with activity against these bacteria. Of the 44 antibacterials under study for development, currently only 15 have some type of action against Gram-negative and only five have progressed to a phase 3 clinical study, but all of these are substances resulting from molecular modifications of drugs already on the market [5,6]. Health authorities around the world are looking for solutions to this crisis and emphasize that we urgently need to develop new alternatives for the treatment of these infections [7,8].

The amide group is widely found and is of fundamental importance for the biological and material properties of a vast number of compounds created by man and present in nature [9]. As Aschale [10] explains, there has been considerable interest in making substitutions for acetanilide derivatives due to this group being present in several synthetic drugs related to a wide range of biological activities, such as antibacterial, antiviral, antifungal, antihelmintic, analgesic, and anti- inflammatory activities.

The acetamides are widely used as herbicides in agriculture, showing potent activity against pests [11]. There are some reports of acetamide derivatives as antimicrobial agents. Katke, Amrutkar, and Khairnar [12] demonstrated excellent antibacterial and antifungal activity for these compounds, Patel et al. [13] synthesized an acetamide from coumarin and obtained a product with good activity against several strains, including *Mycobacterium tuberculosis*. Jetti, Chidurala, and Meshram [14] demonstrated that the combination of an acetamide with monobactamic antibiotic showed good activity against Gram-positive and negative.

In the acetamide group, the presence of the chloro atom seems to improve the antimicrobial activity of these molecules. In a study by Bravo et al. [15] the antimicrobial activity of *N*-(2-hydroxyphenyl) acetamide against *Candida albicans* was evaluated. As a result, the absence of activity against this microorganism was observed. On the other hand, the same substance with the addition of a chloro atom (2-chloro-*N*-(2-hydroxyphenyl) acetamide) was able to inhibit 96.6% of *C. albicans* strains. Thus, the addition of chlorine to the alpha carbon caused the molecule to show activity against these yeasts.

The *N*-(4-fluoro-3-nitrophenyl)acetamide (**A1**) and 2-chloro-*N*-(4-fluoro-3-nitrophenyl)acetamide (**A2**) are substances commonly used as reaction intermediates [16,17], about which there are still no reports in the scientific literature on their biological activities. In view of the antimicrobial potential of acetamides and chloroacetamides, and the urgent need to develop new therapeutic alternatives for the treatment of infections caused by *K. pneumoniae*, this study aimed to investigate the antibacterial activity of these molecules on *K. pneumoniae*, evaluating whether the presence of the chloro atom improves this effect. Then, the most promising molecule was investigated, analyzing its antibacterial action more thoroughly, as well as its cytotoxic and pharmacokinetic profile, in order to contribute to future studies for the viability of a new antibacterial drug.

## 2. Results and Discussion

### 2.1. Chemistry

**Figure 1:**
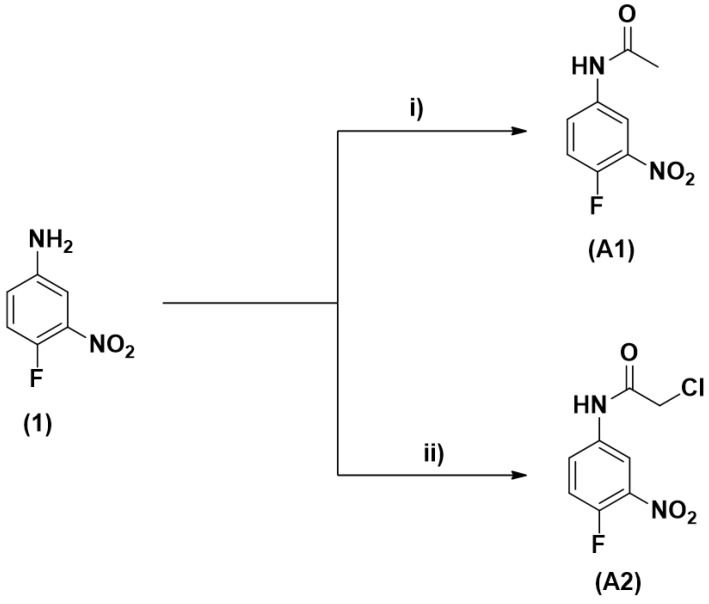
Synthetic route for the synthesis of the target molecules. (**A1**): *N*-(4-fluoro-3-nitrophenyl) acetamide, (**A2**): 2-chloro-*N*-(4-fluoro-3-nitrophenyl)acetamide. Reagents and conditions: (i) Ac_2_O, rt, 24 h, 85%; (ii) 2-chloroacetyl chloride, Et_3_N, DCM, 0 °C to rt, 20 h, 80%.

The target molecules **A1** and **A2** were synthesized using specific acylating agents for each one (Figure 1). The compound *N*-(4-fluoro-3-nitrophenyl)acetamide (**A1**) was obtained with 85% yield from the acetylation reaction of 4-fluoro-3-nitroaniline with acetic anhydride at room temperature [16]. The compound 2-chloro-*N*-(4-fluoro-3-nitrophenyl)acetamide (**A2**) was obtained from the reaction between 2-chloroacetyl chloride and 4-fluoro-3-nitroaniline in the presence of triethyamine (Et_3_N), with a yield of 80% [17]. All compounds were purified using the recrystallization method and their purities were confirmed by the melting point.

The structures of the compounds **A1** and **A2** were characterized by infrared (IR), ^1^H and ^13^C nuclear magnetic resonance (NMR) spectroscopy (Supplementary Materials). In the hydrogen spectra of the compounds, the proton signal attributed to N-H can be observed in the region between 8.47 and 10.37 ppm. For compound **A1**, a signal assigned to three methyl protons in the form of a singlet referring to the acetyl group is observed at 2.07 ppm. In the spectrum of compound **A2** was observed a referring singlet for two methylenic protons (CH_2_) at 4.22 ppm. In all compounds, aromatic protons are in the range of 7.29–8.48 ppm. In the carbon spectra of the synthesized compounds, we observed the characteristic signal of amide carbonyl in the region of 164.53–168.97 ppm. In the spectrum of compound **A1** the signal at 23.89 ppm refers to the methyl carbon of the acetyl group. In compound **A2** a signal referring to methylene carbon (CH_2_) at 42.81 ppm was observed. In all compounds, the signals attributed to aromatic carbons were found at 115.27–152.44 ppm.

In the infrared (IR) spectra of all compounds, we can observe stretches of approximately 1678 cm^−1^ referring to the secondary amide carbonyl. For all compounds, the N-H stretching occurred around 3300 cm^−1^. Folding bands of N−H for all compounds showed absorption values close to 1610 cm^−1^. The asymmetric and symmetrical stretches related to the NO_2_ group of all molecules showed absorption values in the region of 1529 cm^−1^ and 1332–1338 cm^−1^, respectively. The C-F stretches of aromatic compounds occurred around 1238 cm^−1^.

### 2.2. Minimum Inhibitory Concentration (MIC) Determination

Two acetamide derivatives were synthesized: *N*-(4-fluoro-3-nitrophenyl) acetamide (**A1**) and 2-chloro-*N*-(4-fluoro-3-nitrophenyl)acetamide (**A2**) (Figure 1), in order to assess which molecule, among these, has the best antibacterial activity on *K. pneumoniae* and whether the presence of the chloro atom in the molecule is capable of interfering with biological activity. The only structural difference between these molecules is at carbon 2 of the amide portion, where in **A2** there is a chloro atom, and in **A1** this atom is absent.

The previous screening for the antibacterial activity evaluation of the molecules was done by determining the minimum inhibitory concentration (MIC). It was possible to observe that **A1** presented MIC of 1024 µg/mL for 100% of the *K. pneumoniae* strains used in this study, while **A2** resulted in a MIC of 512 µg/mL for the same strains (Table 1).

The results of the antimicrobial activity suggest that the chloro atom is directly related to the potency of the antibacterial activity observed, since the addition of this atom (**A2** substance) made its antibacterial activity twice as potent as that of its precursor (**A1** substance).

A study by Katke, Amrutkar, and Khairnar [12] investigated the antibacterial potential of thirteen different acetamides using the agar diffusion methodology. As results, they observed zones of inhibition ranging between 7 and 30 mm against *Escherichia coli*, 16–35 mm against *Pseudomonas aeruginosa* and 10–36 mm on *Staphylococcus aureus*. Only two of the thirteen substances evaluated did not show antibacterial activity for any strain tested, showing the excellent biological potential of acetamides.

In addition, a study by Bravo et al. [15] showed that the presence of the chloro atom seems to improve the antimicrobial activity of these molecules. The authors evaluated the antimicrobial activity of *N*-(2-hydroxyphenyl) acetamide against *Candida albicans* was evaluated and the substance did not have the ability to inhibit fungal growth. However, the same substance with the addition of a chloro atom (2-chloro-*N*-(2-hydroxyphenyl)acetamide) was able to inhibit 96.6% of *C. albicans* strains. Thus, the addition of chloro to the alpha carbon was essential for the biological activity of the molecule, similarly to the results obtained in this study.

At the present work, the **A1** and **A2** MICs against *K. pneumoniae* show a moderate to weak antibacterial activity of these acetamides. However, it is important to note that there are no reports of antimicrobial activity of these substances and the results indicate their potential applicability for use against bacterial strains, being a starting point for new structural changes to be made, aiming to improve this biological activity. It is interesting that more analyzes are carried out on the action of these substances against other bacterial species, since the results may vary and even be more favorable. Given the current need to discover new antibiotic pharmacological classes, it is interesting that microbiological studies with this group of molecules are further explored.

Due to the **A2** molecule demonstrated greater potential against *K. pneumoniae*, it was selected as the object of study in the present work, to better evaluate its antibacterial and cytotoxic effect.

### 2.3. Molecular Docking Analysis

To understand what is the possible molecular target on which the **A1** and **A2** molecules act to exert the observed antibacterial effect, as well as to better investigate the influence of the chloro atom to anchor the molecule in the active site of the target enzyme, molecular docking analyzes were performed.

The accuracy of molecular docking was validated through re-docking, where it was observed that the RMSD value for the analyzed enzymes remained within the acceptable range 0–2 Å [18]. Table 2 shows the binding energies of the two molecules with the chosen enzymes. The **A2** substance showed better binding energies compared to **A1,** for all tested enzymes, showing that the chloro helps the molecule to have a more effective anchoring in different active sites.

**Figure 2:**
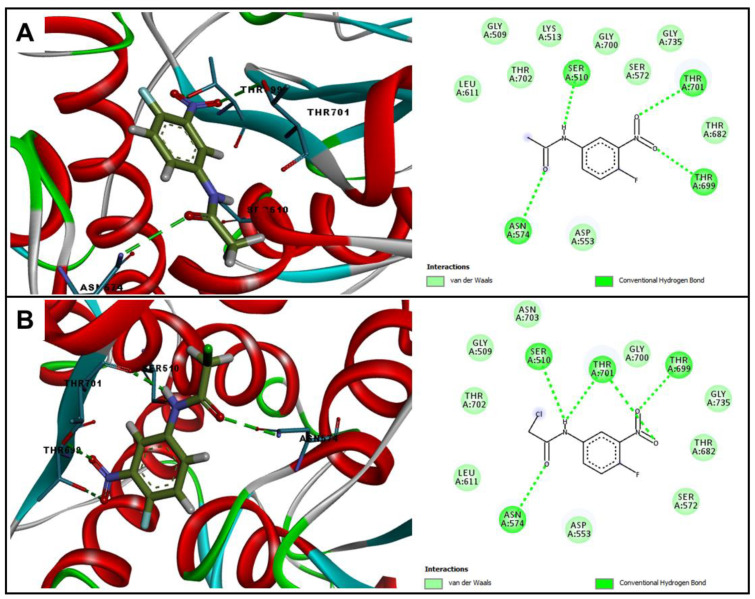
Two and three-dimensional representation of interactions performed by (**A**) **A1** and (**B**) **A2** at the PBP1 (5HLA) active site.

Among all tested enzymes, **A2** showed better binding energies for the enzymes penicillin binding proteins (PBPs) 1 and 3, with −59.0 kcal∙mol^−1^ for both enzymes. In addition to the values of binding energy, another parameter of extreme importance is the interactions carried out with the active site of the enzyme. With PBP1, the **A2** and **A1** molecules performed hydrogen bonding interactions with the Thr701, Thr 699, Asn574, and Ser510 residues, together with several Van der Walls interactions with other residues from the active site, as can be seen in Figure 2. However, the presence of chloro in **A2** is fundamental to better stabilize this molecule in the active site of the enzyme, due to Van der Walls interactions with the Ser510 and Thr702 residues that are important for a good anchorage in this active site [19]. These differences in anchoring reflect the potency of the antibacterial activity that was observed previously, confirming that the **A2** molecule has a more pronounced antibacterial effect.

Thus, the results suggest that the **A2** substance acts on the PBP enzyme. This enzyme is responsible for maintaining the peptidoglycan layer of the bacterial cell wall, it acts by cross-linking the adjacent peptide chains of immature peptidoglycan units. Since it plays a fundamental role in the survival of the bacterial cell, this enzyme is the target of many antibacterial drugs, especially β-lactams. Molecules that act by inhibiting cell wall synthesis can lead to changes in cell shape and size, induction of responses to cell stress and, ultimately, cell lysis [20].

### 2.4. Minimum Bactericidal Concentration (MBC) Determination

In order to better identify the mode of action of the **A2** substance, the minimum bactericidal concentration (MBC) was determined. As a result, it was observed that the MBC was 512 µg/mL for all *K. pneumoniae* strains used in this study, as described in Table 3.

A bactericidal substance is capable of killing the bacterial cell, while bacteriostatic substances inhibit or slow its growth without causing death. A drug is considered to exhibit bactericidal activity when the ratio between MBC and MIC is ≤4 [21,22]. Thus, since the MIC of **A2** was 512 µg/mL and the MBC was also 512 µg/mL for all *K. pneumoniae* strains, there is a 1:1 proportion. Therefore, the substance **A2** acted in a bactericidal manner, from the MIC.

Data on the classification of a given substance as bacteriostatic or bactericidal can provide valuable information on the potential action of antibacterial agents in vitro. However, these concepts are applicable to experiments in standardized conditions in vitro and can vary according to the type of bacteria, amount of inoculum and duration of the test. These variations are, therefore, observed in clinical practice, where the conditions found are as varied as possible [22]. Thus, it is necessary to combine this information with the pharmacokinetic and pharmacodynamic data of this acetamide, to provide a more significant prediction of in vivo efficacy.

### 2.5. Time-Kill Kinetics Against K. pneumoniae

**Figure 3:**
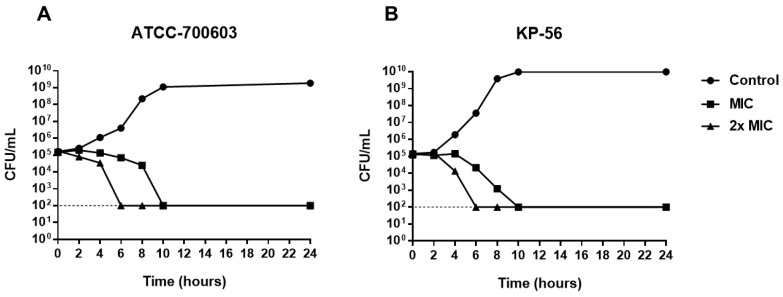
Time-kill curve for *K. pneumoniae* (**A**) ATCC-700603 and (**B**) KP-56 treated with **A2** at MIC and 2× MIC concentrations.

To assess the time of action of **A2** on *K. pneumoniae*, time-kill kinetics was performed, evaluating the number of colony-forming units (CFU) over 24 h of cell exposure to the substance. As shown in Figure 3, after 10 h of treatment with **A2** it is possible to observe the total reduction of viable cells. The same effect is observed after 6 h in the presence of **A2** in the concentration corresponding to 2× MIC. Bactericidal activity was defined as a ≥3-log reduction in bacterial counts (log_10_ CFU/mL) [23], therefore, these results confirm the data observed in the determination of the minimum bactericidal concentration, proving that **A2** acts as a bactericide agent, from the MIC.

Although **A2** MIC and 2× MIC are bactericidal, the time-kill kinetics is reduced with increasing concentration, as observed in the dead time curve (Figure 3). Thus, the higher the concentrations of A2 substance, the shorter the time required to obtain the bactericidal effect.

### 2.6. Effect on Bacterial Cell Integrity

The analysis of molecular docking suggests that **A2** acts by inhibiting the PBP enzyme, responsible for maintaining the bacterial cell wall. Agents acting on this target cause a series of destabilizations, which result in cell lysis [20] and consequent release of cytoplasmic content. In these cases, cytoplasmic ions such as potassium and phosphate are first released to the external environment, followed by larger molecules such as deoxyribonucleic acid (DNA) and ribonucleic acid (RNA), in addition to other materials. The nucleotides have a strong absorption at 260 nm and, therefore, it is possible to measure cell lysis through UV absorption of the supernatant of cells treated with the antibacterial substance [24,25].

**Figure 4:**
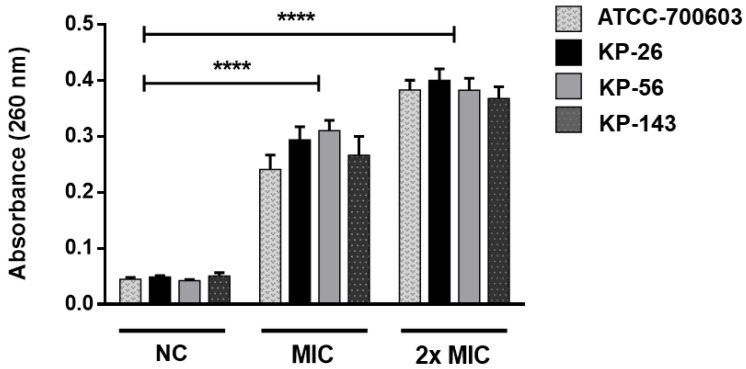
The effect of **A2** on bacterial cell integrity. Release of 260 nm absorbing material from *K. pneumoniae* suspensions treated with MIC and 2× MIC of **A2** for 10 h and 6 h, respectively. Statistical analysis compared to negative controls (NC): **** *p* ≤ 0.0001.

The Figure 4 shows the results when *K. pneumoniae* is treated with different concentrations of **A2** (MIC and 2× MIC) compared to untreated cells (negative control). After 10 h of treatment with **A2** at MIC, there is a significant release of cytoplasmic contents. Cell lysis is also evident after six hours of **A2** action at a concentration corresponding to 2× MIC.

The results indicated that irreversible damage to *K. pneumoniae* cells can occur, leading to the loss of cellular constituents, such as nucleic acid and some essential molecules, causing bacterial lysis. These results reinforce the previous data, confirming the bactericidal effect of **A2** and its possible action on PBP enzyme.

### 2.7. Hemolytic Activity

The toxicity of bioactive substances must be assessed to ensure less toxic effect on the system to which it is intended for therapeutic application. In this context, tests with in vitro cytotoxicity screenings are a good alternative to assess the toxic potential of substances. In a study by Rangel et al. [26], red blood cells were used to determine the toxicity of promising drugs. Its effect was classified by percentage considering: low (0% and 40%), moderate (40% and 80%), and high above (80%).

**Figure 5:**
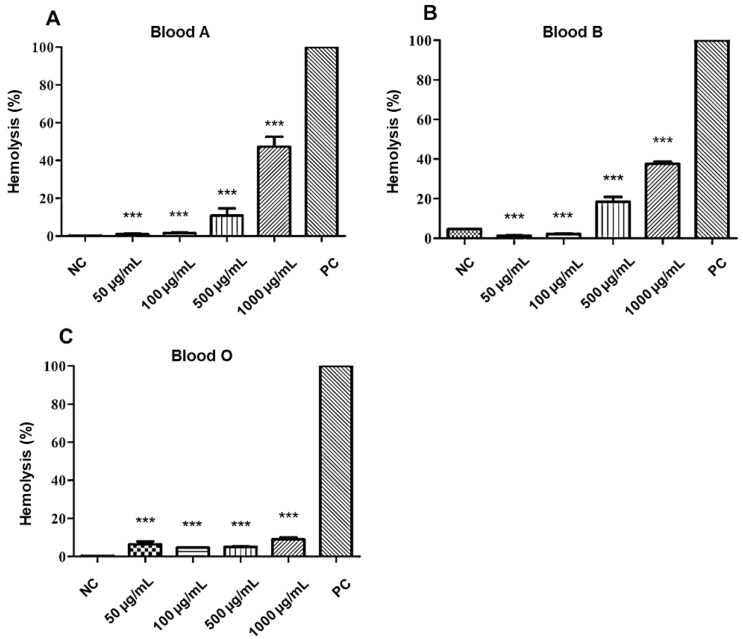
Cytotoxic effect of **A2** on red blood cells (RBC) of the different blood types of the ABO system: (**A**) type A, (**B**) type B, and (**C**) type O. NC: negative control. PC: positive control. Statistical analysis compared to positive control: *** *p* ≤ 0.001.

The analysis of the hemolytic potential of the **A2** molecule showed a low hemolysis, being approximately 4–20% at 50–500 µg/mL concentrations, in all tested blood types. The highest concentration of the tested molecule (1000 µg/mL) against the erythrocytes of the ABO system demonstrated a percentage of lysis that varied according to blood type. On types A and B, moderate hemolysis was obtained, ranging from 37–47%. However, for type O, a low percentage of hemolysis was observed, with an average of 8% of erythrocyte lysis, as can be seen in Figure 5.

The results suggest that **A2** molecule has less potential for hemolysis in the absence of surface carbohydrates from red cell membranes, such as *N*-acetylgalactosamine (serotype A) and d-galactose (serotype B), since the assay revealed a better interaction of the molecule in order O < B < A blood types.

Other acetamide derivatives with significant biological activity also demonstrate low cytotoxic potential. In studies carried out by Gentile and Calabrese [27] evaluating the in vitro toxicity of acetamide-derived molecules with potential herbicide, the production of oxidative stress against the erythrocytes of rats, sheep, and dogs was not observed. The test describes, in addition to low levels of hemolysis, the reduction in oxidative stress of the test on the formation of MetHb. Likewise, in the present study, the results indicate that the **A2** molecule has a low toxicological effect when tested in human red blood cells.

### 2.8. Antihemolytic Activity

**Figure 6:**
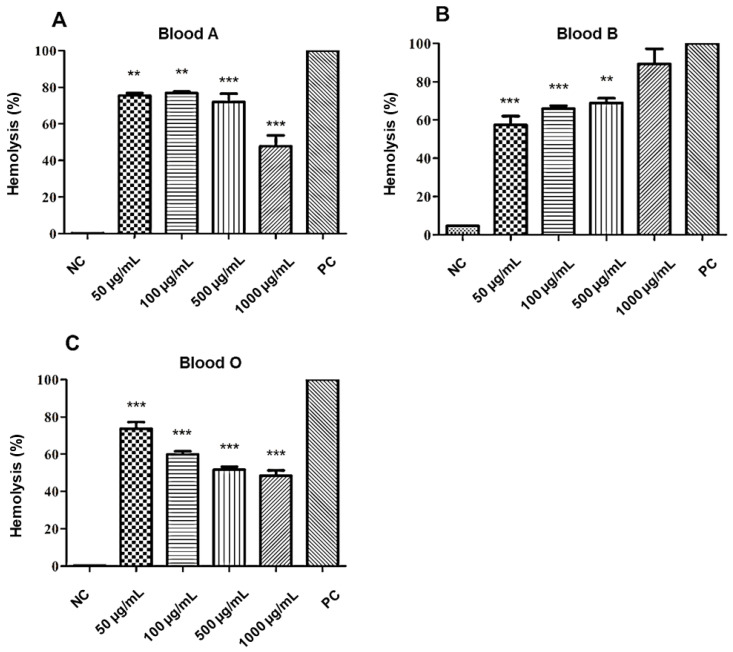
Anti-hemolytic effect of **A2** on red blood cells (RBC) of the different blood types of the ABO system: (**A**) type A, (**B**) type B, and (**C**) type O. NC: negative control. PC: positive control. Statistical analysis compared to positive control: *** *p* ≤ 0.001; ** *p* ≤ 0.01.

The anti-hemolytic evaluation of the **A2** molecule showed a percentage of hemolysis that is classified as moderate (>40% and <80%) [26], for all concentrations evaluated in the three blood types of the ABO system, except for the concentration of (1000 µg/mL), which presented hemolysis (>80%) in type B red blood cells. The osmotic protection performance was also perceived with an increase in the concentration of the tested substance, where, the higher the concentration tested, the better the red cell protection effect, as can be seen in Figure 6.

The concentration of 500 µg/mL of the **A2** molecule revealed a moderate potential to promote the stability and protection of the erythrocytes of the ABO system. However, there was a variation in lysis according to the blood type and concentration of the substance evaluated, in the following order O < A < B.

According to a study by Autore et al. [28] acetamide derivatives may have antioxidant and anti-inflammatory activity, revealing low toxicity potential in tests with liver cell lines. In this present study, the anti-hemolytic test revealed a moderate percentage of lysis, suggesting a higher concentration of the **A2** molecule to achieve good levels of protection for red blood cells. However, more studies need to be carried out to better investigate this protective activity.

### 2.9. Mutagenic Effect on Oral Mucosa Cells

In vitro genotoxicity studies evaluate the cytotoxic capacity of substances on certain cell types isolated in liquid media. The possible action of toxicity is revealed through the damage caused to the nucleus by the time of exposure to test substances. Cytotoxic effects can be verified by suggestive nuclear alterations, such as: micronucleus, binucleation, karyolysis, and cariorrexe [29].

The **A2** genotoxicity test showed the appearance of few cellular alterations compatible with toxic damage. The toxic findings were more present in the groups exposed to the H_2_O_2_ solution (positive control). The cells treated with different concentrations of A2 showed few cellular changes compared to the positive control, as can be seen in Table 4.

The data reveal that **A2** showed low toxicity, with the presence of normal cells in amounts greater than 80% in all tested concentrations. Among the nuclear alterations found, karyolysis, and karyorrhexis were identified in greater quantities, although these indices are lower than those found in the positive control, suggesting a safe threshold of low toxicity of the **A2** molecule on the oral mucosa cells.

Other studies, such as the one carried out by Moore et al. [30], point out that some acetamides have low cytotoxic, clastogenic, aneugenic, or mutagenic potential, which reinforces the data found in this work. However, in order to better evaluate the possibility of using A2 and its derivatives as an antibacterial drug, further studies must be performed to ensure the safety of in vivo use.

### 2.10. In Silico Analysis of Pharmacokinetic Parameters

In this work, the physical-chemical parameters of the A2 molecule were obtained through an in silico approach using the free online software SwissADME to verify its theoretical absorption. According to Table 5, the physical-chemical parameters analyzed according to the rules of Lipinski [31], Ghose [32], Veber [33], and Egan [34] it is possible to predict whether the molecule under study has the potential to be developed as a drug.

The in silico approach showed that the substance **A2** presents values of physicochemical parameters within the acceptable region based on the rules of Lipinski [31], Ghose [32], Veber [33], and Egan [34]. The hydrogen acceptors calculated was 4 and that of hydrogen donors was 1 and are in accordance with Lipinski′s rule. To develop a molecule with the potential for a particular drug, the molecule must be able to cross plasma membranes. Thus, the partition coefficient of a substance in n-octanol and water (Log P), is an important parameter. According to the rules of Lipinski (Log P_o/w_ ≤ 5), Ghose (Log P_o/w_ ≤ 5.6), and Egan (Log P_o/w_ ≤ 5.8) the **A2** molecule fits the required conditions imposed, as its Log P is 1.33.

Another important factor for the development of a compound is the molar mass, since it is related to the transport of the drug within the body. The molecule under study has a molar mass of 232.60 g/mol, which meets the criteria of Lipinski and Ghose. Other important criteria are the number of rotatable connections and the value of the TPSA. According to Veber, molecules that have a number of rotatable bonds ≤ 10 and TPSA values ≤ 140 Å, indicate that there is a high probability of being administered orally. According to Table 5, the molecule showed values of 4 for the number of rotatable bonds and a TPSA of 74.92 Å2. As for solubility (Log S), it considers an important quality of a drug for the absorption and distribution of the molecule in the body. According to the class presented in Table 5, substance **A2** is soluble, as it presented the Log S (coefficient of solubility determined by the ESOL method) with a value of −2.30.

Based on the above, in silico results show a favorable pharmacokinetic profile for **A2** substance. Inadequate penetration of the infection site is one of the main factors related to the failure of antibacterial therapies. The active drug needs to reach the bacteria in appropriate body fluids and tissues at concentrations necessary to kill or suppress pathogens’ growth. Even for substances with good in vitro activity, the pharmacokinetic parameters are decisive for its clinical use [22]. Thus, **A2** presents characteristics that suggest a good drug candidate and can display a high prospect of being used orally, with excellent theoretical oral bioavailability and good solubility, which can guarantee adequate absorption and distribution in vivo.

## 3. Materials and Methods

### 3.1. Chemistry

The reagents 4-fluro-3-nitroaniline, triethylamine (Et_3_N), acetic anhydride, chloroacetyl chloride, chloroform, and ethanol were purchased from Merck/Sigma-Aldrich^®^ (Darmstadt, Germany). The recrystallization technique was used to purify the compound and its purity was confirmed from the melting point measurement using the MQAPF-302 (Microquímica, São Paulo, Brazil) apparatus. A Shimadzu spectrometer model IRPrestige-21 FT-IR (Barueri, São Paulo, Brazil) with an attenuated total reflection (ATR) accessory produced the infrared (IR) spectrum. The hydrogen and carbon nuclear magnetic resonance (NMR) spectra were obtained from a Bruker Avance 400 apparatus. The hydrogen spectra (^1^H) were obtained at the frequency of 400 and 500 MHz using deuterated solvents chloroform (CDCl_3_) and dimethylsulfoxide (DMSO). The carbon spectra (^13^C) were obtained at the frequency of 101 and 126 MHz using deuterated solvents chloroform (CDCl_3_) and dimethylsulfoxide (DMSO). The chemical distances (δ) were generated in parts per million (ppm) and in Hertz (Hz) the coupling constants (*J*) were determined.

### 3.2. Preparation of N-(4-fluoro-3-nitrophenyl)acetamide (A1)

The 4-fluro-3-nitroaniline (3.12 g, 20 mmol) is added, in small portions, in a 50 mL flask containing acetic anhydride (11 mL, 116 mmol) under constant stirring at room temperature. The reaction mixture is stirred for 12 h. At the end of the reaction, ice water is added and the reaction mixture was stirred for 30 min. The precipitate formed was filtered, washed with water and dried. The product was recrystallized from an ethanol/water mixture (8:2). Yield: 85%, brown solid. m.p.: 142–144 °C (Lit. [16]: 140–141 °C). IV (ATR): ν/cm^−1^ 3356 (N-H), 3124, 3064 (C-HAr), 1678 (C=O), 1610 (N-H), 1529 and 1332 (NO_2_), 1482 (C=C), 1238 (CAr-F), 1155, 1141 (C-HAr), 833 (C-N for ArNO2). 1H NMR (400 MHz, DMSO-*d*_6_) δ 10.37 (s, 1H, NH), 8.48 (dd, *J* = 6.9, 2.7 Hz, 1H, CHAr), 7.81 (ddd, *J* = 9.1, 3.9, 2.8 Hz, 1H, CHAr), 7.50 (dd, *J* = 11.2, 9.1 Hz, 1H, CHAr), 2.07 (s, 3H, CH3). 13C NMR (101 MHz, DMSO) δ 168.97, 150.19 (*J* = 257.8 Hz), 136.31 *(J* = 7.8 Hz), 136.05 (*J* = 3.3 Hz), 126.23 (*J* = 8.1 Hz), 118.75 (*J* = 21.8 Hz), 115.27 *(J* = 3.3 Hz), 23.89 [16].

### 3.3. Preparation of 2-Chloro-N-(4-fluoro-3-nitrophenyl)acetamide (A2)

The chloroacetyl chloride (2.71 g, 24 mmol) is added slowly in a 50 mL flask containing a mixture of 4-fluoro-3-nitroaniline (1) (3.12 g, 20 mmol) and Et_3_N (3, 3 mL, 0.024 moles) solubilized in 20 mL of CHCl_3_ at 0 °C in an ice bath. At the end of the addition of chloroacetyl chloride, the ice bath is removed and the reaction is stirred at room temperature. After 20 h of reaction, the reaction mixture was subjected to extraction. The organic phase is washed with water (3 × 30 mL), brine (3 × 30 mL), dried over anhydrous sodium sulfate and then concentrated under reduced pressure, providing the precipitate. The material was purified using a mixture of ethanol/water (9:1) in a recrystallization process. Yield: 80%, brown solid, m.p.: 91–93 °C (Lit. [17]: 90–92 °C). IV (ATR): ν/cm^−1^ 3332 (N-H), 3132, 3074 (C-H_Ar_), 2943 (C-H_Aliph_), 1678 (C=O), 1606 (N-H), 1529 and 1338 (NO_2_), 1492 (C=C), 1228 (C_Ar_-F), 831 (C-N for ArNO_2_). ^1^H-NMR (500 MHz, CDCl_3_) *δ* 8.47 (s, 1H, NH), 8.32 (dd, *J* = 6.4, 2.8 Hz, 1H, CH_Ar_), 7.89–7.85 (m, 1H, CH_Ar_), 7.29 (dd, *J* = 10.1, 9.1 Hz, 1H, CH_Ar_), 4.22 (s, 2H, CH_2_). ^13^C-NMR (126 MHz, CDCl_3_) *δ* 164.53, 152.43 (*J* = 263.6 Hz), 133.44, 133.41, 127.13 (*J* = 8.1 Hz), 119.13 (*J* = 22.1 Hz), 117.55 (*J* = 2.9 Hz), 42.81 [17].

### 3.4. Strains

The clinical isolates strains of *K. pneumoniae* were used in this study belong to the MICOTECA of the Antibacterial and Antifungal Activity Research Laboratory of the Federal University of Paraíba, Brazil, which are: KP-25, KP-26, KP-56, KP-83, KP-87, KP-138, KP-143, KP-166, KP-173, KP-176, KP-260, and KP-326. The American Type Culture Collection strain ATCC-700603 was used as controls. For use in the assays, bacterial suspensions were prepared in 0.9% saline solution, from fresh cultures, and adjusted to the McFarland standard 0.5 scale.

### 3.5. Minimum Inhibitory Concentration (MIC)

To assess the antibacterial activity of substances **A1** and **A2**, the minimum inhibitory concentration against *K. pneumoniae* strains was determined. The substances were solubilized in dimethyl sulfoxide (DMSO) at 5% and Tween-80 at 2%, to obtain emulsions in the concentrations necessary for use in the tests. The MIC determination was performed using the broth microdilution technique in a 96-well plate to obtain different concentrations of **A1** and **A2** [35]. In parallel, controls for sterility, cell viability, and interference of the vehicles used in the preparation of substance emulsions (DMSO and Tween-80) were also performed. MIC is defined as the lowest concentration capable of causing complete inhibition of bacterial growth after 24 h at 35 ± 2 °C.

### 3.6. Molecular Docking

The chemical structures of the compounds were designed using the software MarvinSketch 18.5, their energies minimized in the program Hyperchem v. 8.0.3, using the molecular mechanics method (MM +) and the semi-empirical method AM1 (Austin Model 1) [36]. The enzymes were obtained from the Protein Data Bank (https://www.rcsb.org/) together with their co-crystallized inhibitors, with PDB ID: 2VF52 (2.9 Å) [37], 5V3D3 (1.54 Å) [38], 5HLA4 (1.7 Å) [19], 2ZD85 (1.05 Å) [39], 3PBS6 (2.00 Å) [40], 6C3U7 (1.85 Å) [41], 1AJ68 (2.30 Å) [42], 1S149 (2.00 Å) [43]. Molecular docking was performed at Molegro Virtual Docker (MVD) software (v 6.0.1, Molegro ApS, Aarhus, Denmark), using the standard parameters of the software, the water molecules were removed and a template was generated in the co-crystallized inhibitor of the PDB.

### 3.7. Minimum Bactericidal Concentration (MBC)

After MIC reading, the **A2** minimum bactericidal concentration was determined by removing aliquots from the microdilution plates in the wells corresponding to concentrations equivalent to MIC, 2× MIC, 4× MIC, and 8× MIC and inoculating in new plates containing only culture broth. All controls were performed in parallel. MBC is defined as the lowest concentration capable of causing complete inhibition of bacterial growth after 24 h at 35 ± 2 °C [21,44].

### 3.8. Time-Kill Kinetics

The determination of the action time of **A2** substance against *K. pneumoniae* (ATCC-700603 and KP-56) was carried out exposing the microorganisms to concentrations equivalent to MIC and 2× MIC during times 0, 2, 4, 6, 8, 10, and 24 h. After incubation on Mueller–Hinton agar, bacterial colonies were counted. Bactericidal activity was defined as a ≥3-log reduction in bacterial counts (log_10_ CFU/mL) [45].

### 3.9. Effect on Bacterial Cell Integrity

The cell integrity can be assessed by releasing intracellular material, mainly nucleotides, which absorb 260 nm wavelengths. The *K pneumoniae* strains ATCC-700603, KP-26, KP-56, and KP-143 were used to perform this test. First, the bacterial inoculum was incubated in BHI broth (1 × 10^7^ CFU/mL) overnight at 35 ± 2 °C. Then, centrifugation was performed at 400× *g* for 10 min. The cell pellet was washed three times with sterile PBS (phosphate-buffered saline) and resuspended in PBS containing the **A2** substance at MIC and 2× MIC concentrations. As a negative control, suspensions that underwent the same procedures, but without treatment with the substance, were used. The tubes corresponding to the MIC were incubated for 10 h at 35 ± 2 °C, and the tubes corresponding to the 2× MIC, for 6 h. After incubation, the suspensions were centrifuged at 400× *g* for 10 min and the supernatants were read on a spectrophotometer at 260 nm [46]. All analyses were performed in triplicate.

### 3.10. Human Erythrocytes and Oral Mucosa Cells Collection

The tests were performed according to the recommendations established by the Code of Ethics of the World Medical Association, after approval by the ethics committee of the Centro Universitário de Patos (number: 3621284). Blood samples (types A, B, and O) and smears of oral mucosa were donated by young, healthy adults.

### 3.11. Hemolytic Activity

The red blood cell (RBC) samples from blood donors were diluted in 0.9% NaCl at a 1:30 ratio and centrifuged at 2500 rpm for five minutes to obtain the 0.5% suspension free of white blood cells and platelets. Then, the **A2** substance was added to the red blood cell suspensions, at 50, 100, 500, and 1000 µg/mL concentrations. As controls, a suspension of red cells without treatment (0% hemolysis) were used as a negative control and a suspension of red cells treated with 1% Triton X-100 (100% hemolysis) as a positive control. The assay was incubated for one hour at 22 ± 2 °C. After that time, the samples were centrifuged at 700× *g* for 5 min and hemolysis was quantified by spectrophotometry at a wavelength of 540 nm [26].

### 3.12. Antihemolytic Activity

The analysis of the osmotic fragility of the red cells was performed using previously prepared red blood cell suspensions at 0.5%. Different concentrations (50, 100, 500, and 1000 µg/mL) of the **A2** substance were used for the treatment of 0.5% RBC suspensions for 1h at 22 ± 2 °C. The samples were centrifuged at 2500 rpm for 5 min and the supernatant was discarded. The erythrocytes were resuspended in a hypotonic solution of 0.24% sodium chloride for one hour at 22 ± 2 °C. After this period, the samples were centrifuged at 700× *g* for 5 min and the hemolysis of the supernatant was quantified by spectrophotometry at 540 nm [47]. As controls, a suspension of untreated red blood cells was used as a negative control (0% hemolysis) and in a hypotonic solution as a positive control (100% hemolysis).

### 3.13. Genotoxic Effects

First, the oral mucosa cells were collected from the donors, which was done with a cyto-brush (smear collector) in the cheek area, and the material obtained was placed in 5 mL of 0.9% NaCl [48]. As controls, cells treated with hydrogen peroxide −0.0005% (positive control) and untreated cells (negative control) were used. The cells were washed twice with saline and centrifuged for 10 min at 400× *g* and kept in saline. After the third wash, the cells were exposed (ex vivo) to **A2** substance at 50, 100, 500, and 1000 µg/mL concentrations for 30 min. The material was centrifuged and the supernatant was discarded before smear preparation. The cells were homogenized in a vortex and placed on the slides at room temperature for drying for 15 min. Then, they were fixed with methanol: acetic acid (3:1) for 15 min and stained with 2% Giemsa. The cells were observed under an optical microscope and about 1000 cells were analyzed on each slide [49,50].

### 3.14. Statistical Analysis

The data were analyzed by the One-way Analysis of Variance (ANOVA) and by the Bonferroni post hoc test, using the GraphPadPrism software (version 6.0 for Windows, San Diego, CA, USA). The data were considered significant when *p* < 0.05.

### 3.15. In Silico Analysis of Pharmacokinetic Parameters

Violations of the rules of Lipinski [31], Ghose [32], Veber [33], and Egan [34] help to evaluate the pharmacokinetic characteristics of drug candidate substances. The following parameters were evaluated about **A2** substance: Physicochemical properties, lipophilicity, water solubility, and druglikeness, using the free online software SwissADME (www.swissadme.ch). Such results were analyzed using the rules of Lipinski [31], Ghose [32], Veber [33], and Egan [34].

## 4. Conclusions

Based on the above, this study showed a good antibacterial potential for acetamides, especially 2-chloro-*N-*(4-fluoro-3-nitrophenyl) acetamide (**A2**), of which chloro is responsible for improving its antibacterial activity, stabilizing the molecule at the site target enzyme. The substance possibly acts on PBP, promoting cell lysis. The analysis of cytotoxicity and mutagenicity show favorable results for future in vivo toxicological tests to be carried out, with the aim of investigating the potential of this molecule. In addition, the substance showed an excellent pharmacokinetic profile, indicating good parameters for oral use. Given the need to discover new antibacterial drugs in order to have alternatives against resistant infections, it is interesting that this substance serves as a starting point for future molecular changes aimed at improving its biological activity.

## Figures and Tables

**Table 1:** Minimum inhibitory concentration (MIC) of **A1** and **A2** against *K. pneumoniae* strains.

*K. pneumoniae* Strains	MIC (µg/mL)	
	A1	A2
ATCC-700603	1024	512
KP-25	1024	512
KP-26	1024	512
KP-56	1024	512
KP-83	1024	512
KP-87	1024	512
KP-138	1024	512
KP-143	1024	512
KP-166	1024	512
KP-173	1024	512
KP-176	1024	512
KP-260	1024	512
KP-326	1024	512

**Table 2:** Binding energies (Moldock score) of PDB enzymes and tested compounds.

Enzyme	Classification	Moldock Score (kcal·mol^−1^)	RMSD (Å)	Moldock Score (kcal·mol^−1^)	
				A1	A2
Glucosamine-6-Phosphate Synthase (2VF5)	Transferase	−89.1	0,18	−52.9	−58.8
FosA (5V3D)	Transferase	−62.3	0,19	−42.6	−46.5
PBP1b (5HLA)	Transferase	−123.4	0,35	−52.2	−59.0
β-lactamase (2ZD8)	Hydrolase	−82.5	0.33	−35.7	−37.3
PBP3 (3PBS)	Hydrolase	−165.5	0.34	−55.6	−59.0
FosAKP (6C3U)	Transferase	−95.2	0.24	−47.5	−55.2
DNA gyrase (1AJ6)	Topoisomerase	−121.3	0.47	−44.0	−50.8
Topoisomerase IV (1S14)	Isomerase	−154.9	0.40	−54.8	−56.8

**Table 3:** Minimum bactericidal concentration (MBC) of **A2** against *K. pneumoniae*.

*K. pneumoniae* Strains	A2	
	MBC (µg/mL)	Effect
ATCC-700603	512	Bactericide
KP-25	512	Bactericide
KP-26	512	Bactericide
KP-56	512	Bactericide
KP-83	512	Bactericide
KP-87	512	Bactericide
KP-138	512	Bactericide
KP-143	512	Bactericide
KP-166	512	Bactericide
KP-173	512	Bactericide
KP-176	512	Bactericide
KP-260	512	Bactericide
KP-326	512	Bactericide

**Table 4:** Genotoxic profile of **A2**.

Group	Karyolysis	Karyorrhexis	Micronucleus	Binucleation	Normal
NC	1.50%	2.00%	2.75%	0.25%	93.50%
PC-H_2_O_2_	7.00%	10.75%	6.50%	1.00%	77.75%
A2-1000 µg/mL	5.00%	7.00%	2.00%	0.50%	83.50%
A2-500 µg/mL	4.00%	5.00%	0.70%	0.50%	86.80%
A2-100 µg/mL	3.00%	4.00%	0.50%	0.00%	92.50%
A2-50 µg/mL	1.00%	1.00%	0.50%	0.00%	97.50%

NC: negative control. PC: positive control.

**Table 5:** In silico studies of Lipinski′s parameters of terpinen-4-ol.

Parameters	A2
Physicochemical Properties	
Formula	C_8_H_6_ClFN_2_O_3_
Molecular Weight	232.60 g/mol
Num. Heavy atoms	15
Fraction Csp3	0.12
Num. Rotatable Bonds	4
Num. H-bonds acceptors	4
Num. H-bonds donors	1
Molar Refractivity	54.33
TPSA^1^	74.92 Å^2^
**Lipophilicity**	
Consensus ^2^ Log P_o/w_^3^	1.33
**Water Solubility**	
Log S (Ali)	−2.30
Class ^4^	Soluble
**Druglikeness**	
Lipinski ^5^	Yes; 0 violation
Ghose ^6^	Yes; 0 violation
Veber ^7^	Yes; 0 violation
Egan ^8^	Yes; 0 violation
Bioavailability Score	0.55

^1^ TPSA: Topological Polar Surface Area; ^2^ Consensus Log P_o/w_ = Average of all five predictions; ^3^ Log P_o/w_ = The partition coefficient between *n*-octanol/water; ^4^ Class = Ali class: insoluble < −10 < poor < −6 < moderately < −4 < soluble < −2 < very < 0 < highly.; ^5^ Lipinski = MM ≤ 500; Log P_o/w_ ≤ 5; H-bond donors ≤ 5; H-bond acceptores ≤ 10;.^6^ Ghose = 180 ≤ MM ≤ 480; 20 ≤ No. of atoms ≤ 70; 40 ≤ Molar Refractivity ≤ 130; -0.4≤ Log P_o/w_ ≤ 5.6; ^7^ Veber = Num. Rotatable Bonds ≤ 10; TPSA ≤ 140 Å^2^; ^8^ Egan = Log P_o/w_ ≤ 5.88; TPSA ≤ 131.6 Å^2^.

## Supplementary Materials

The following are available online. Figure S1. FTIR (ATR) spectrum of A1 substance; Figure S2. 1H NMR spectrum (400 MHz, DMSO-d6) of A1 substance; Figure S3. 13C NMR spectrum (101 MHz, DMSO-d6) of A1 substance; Figure S4. FTIR (ATR) spectrum of A2 substance; Figure 5. 1H NMR spectrum (500 MHz, CDCl3) of A2 substance; Figure S6. 13C NMR spectrum (126 MHz, CDCl3) of A2 substance.

## Abbreviations

ATCC	American Type Culture Collection
BHI	Cerebral Heart Infusion (Broth)
CFU	Colony Forming Units
DMSO	Dimethyl Sulfoxide
MBC	Minimum Bactericidal Concentration
MIC	Minimum Inhibitory Concentration
MVD	Molegro Virtual Docker
MW	Molecular Weight
NC	Negative control
PBS	Phosphate Buffered Saline
PC	Positive control
PDB	Protein Data Bank
RBC	Red Blood Cell
RMSD	Root Mean Standard Deviation
TPSA	Topological polar surface area
