# Supplementary material for: Potential of 2-Chloro-N-(4-fluoro-3-nitrophenyl)acetamide Against Klebsiella pneumoniae and In Vitro Toxicity Analysis
Source: Molecules. 2020 Aug 31;25(17):3959. doi: 10.3390/molecules25173959 (PMC7504751; doi:10.3390/molecules25173959)
Supplement: Supplementary file 1 [file molecules-25-03959-s001.pdf]

molecules

MDPI

Supplementary Material

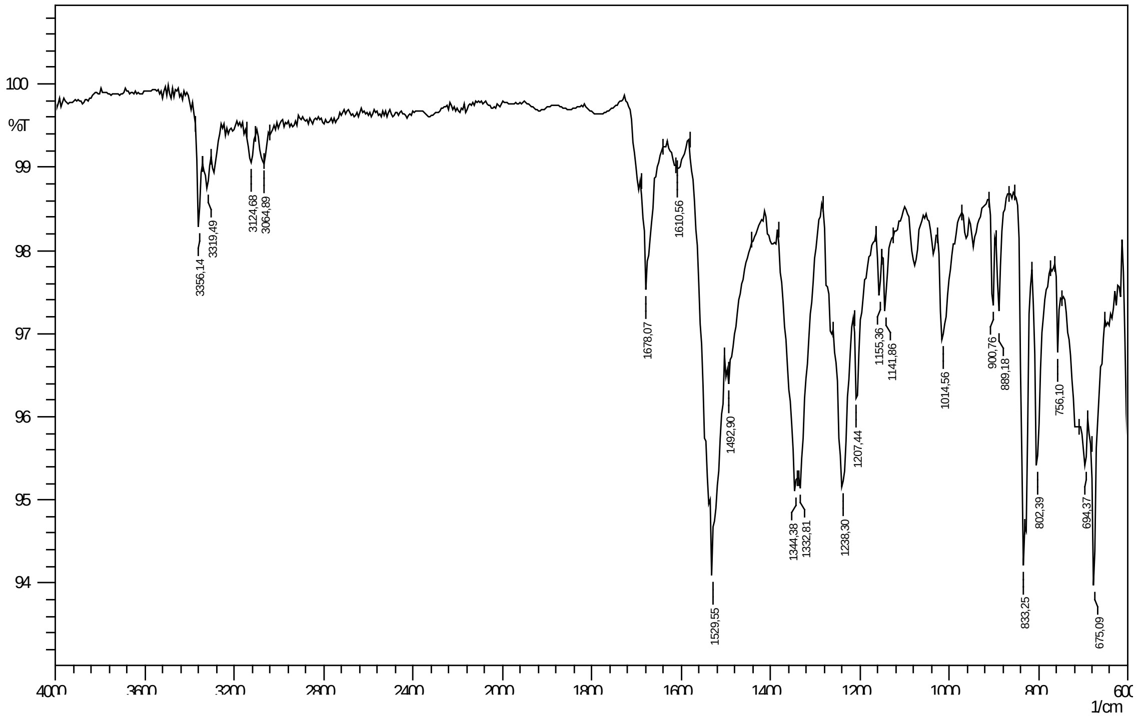

Figure S1. FTIR (ATR) spectrum of A1 substance.

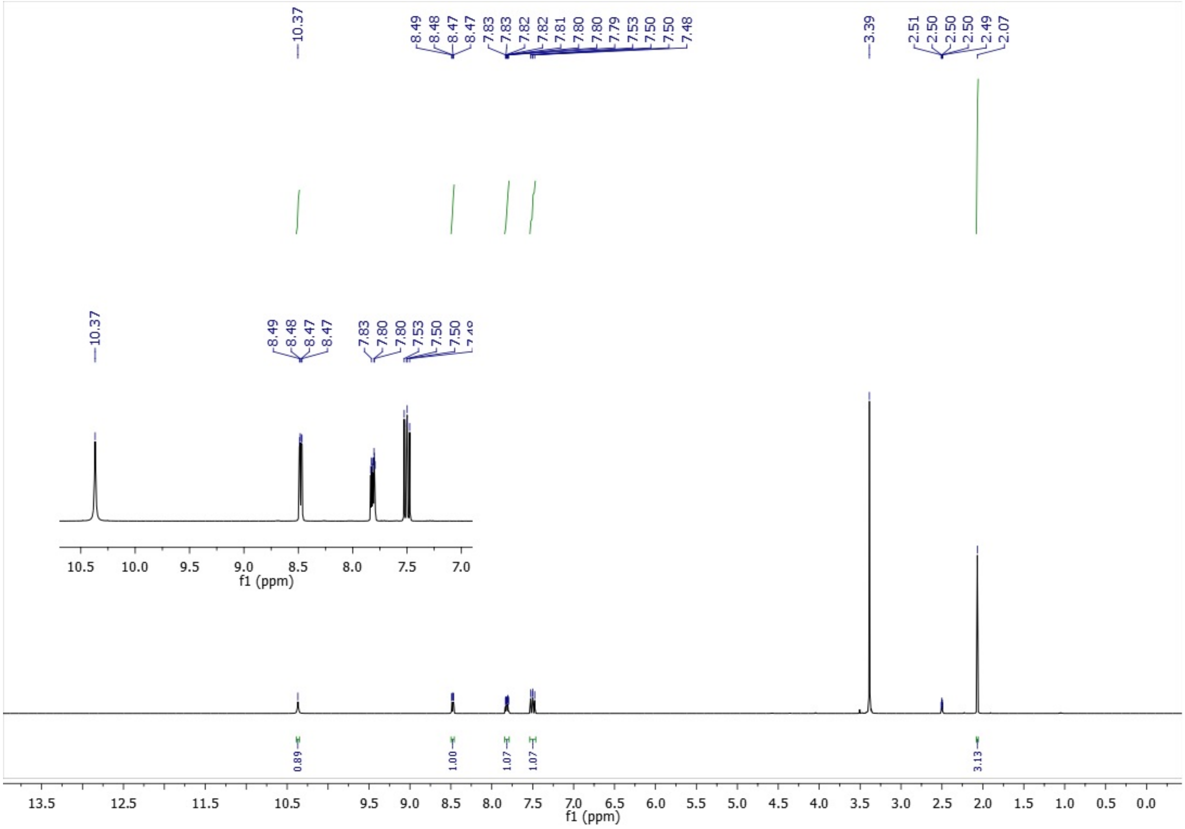

Figure S2. ^{1}H NMR spectrum (400 MHz, DMSO-d_6) of A1 substance.

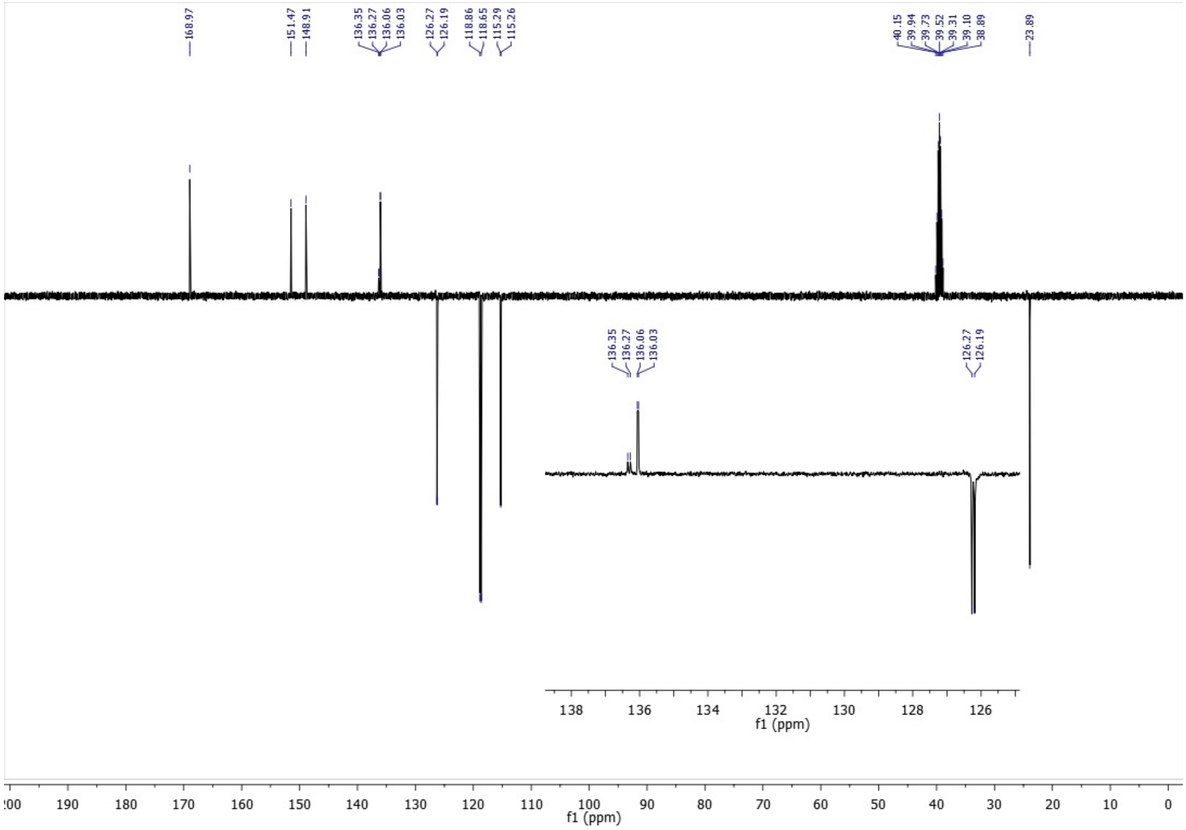

Figure S3. ^{13}C NMR spectrum (101 MHz, DMSO-d_6) of A1 substance**.**

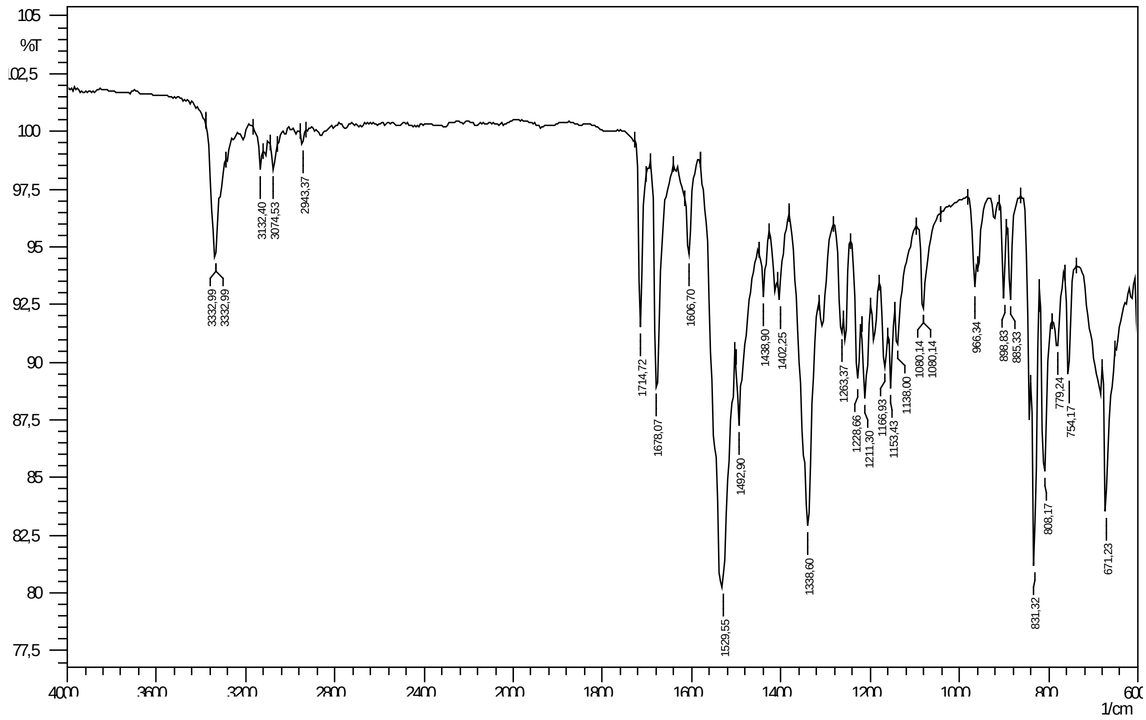

Figure S4. FTIR (ATR) spectrum of A2 substance**.**

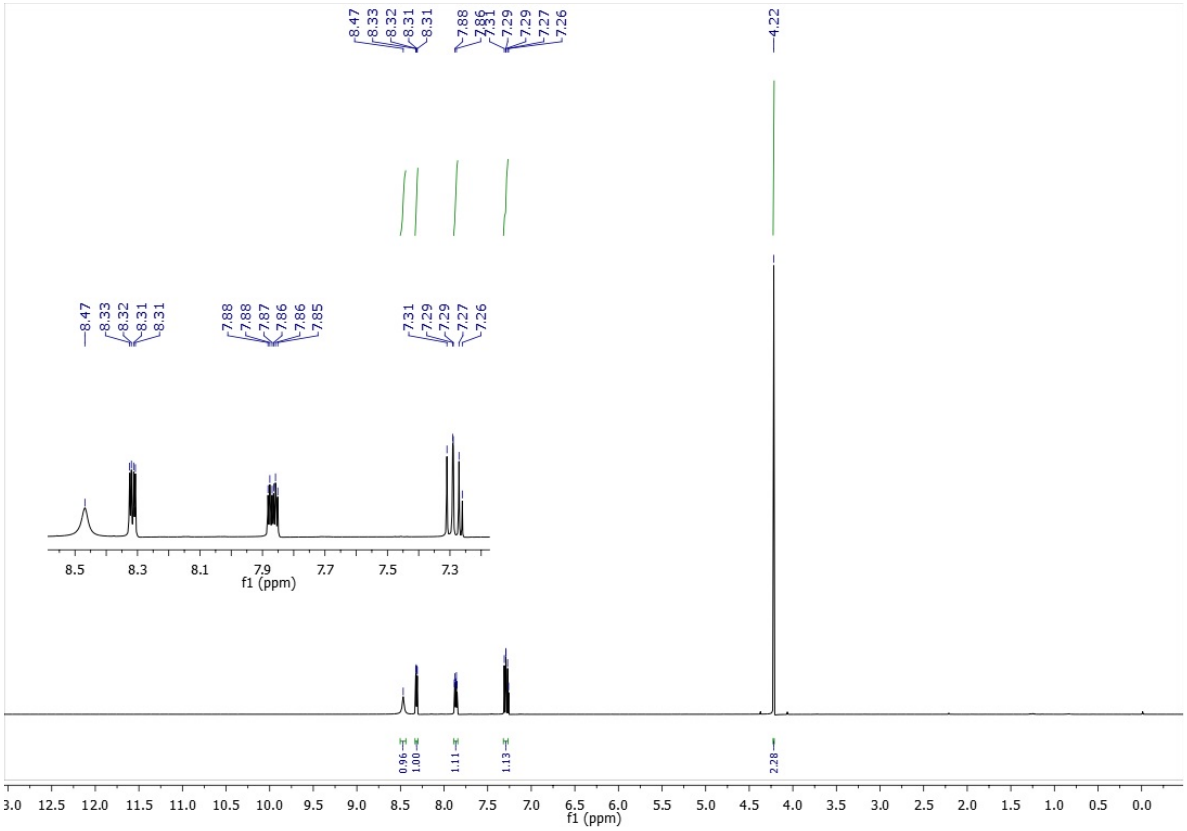

Figure S5. ^{1}H NMR spectrum (500 MHz, $CDCl_3$) of A2 substance**.**

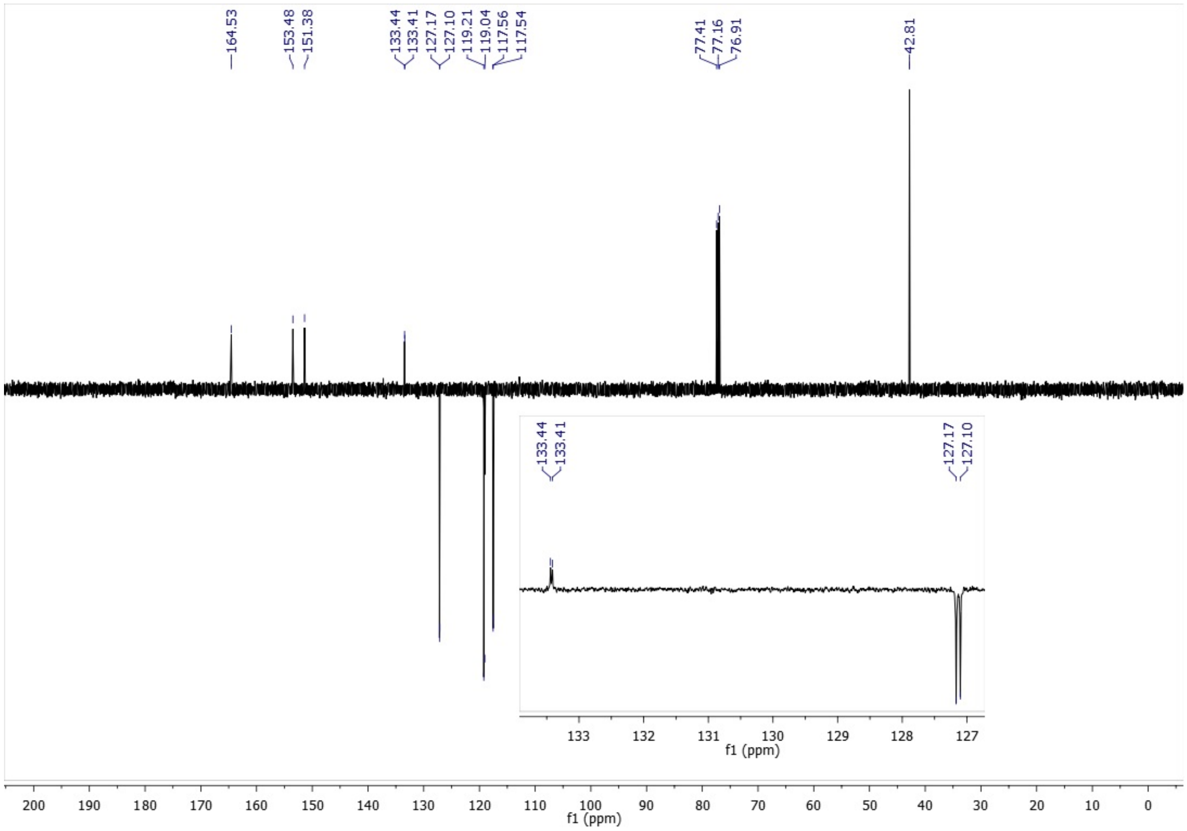

Figure S6. ^{13}C NMR spectrum (126 MHz, $CDCl_3$) of A2 substance**.**
